# Collagen biosynthesis stimulation and anti-melanogenesis of bambara groundnut (*Vigna subterranea*) extracts

**DOI:** 10.1080/13880209.2020.1822419

**Published:** 2020-09-30

**Authors:** Romchat Chutoprapat, Waraporn Malilas, Rattikarl Rakkaew, Sarinporn Udompong, Korawinwich Boonpisuttinant

**Affiliations:** a Department of Pharmaceutics and Industrial Pharmacy, Faculty of Pharmaceutical Sciences, Chulalongkorn University, Bangkok, Thailand; b Institute of Nutrition, Mahidol University, Nakhon Pathom, Thailand; c Innovative Natural Products from Thai wisdom (INPTW), Thai Traditional Medicine College, Rajamangala University of Technology Thanyaburi, Pathumthani, Thailand

**Keywords:** Antioxidation, anti-ageing, whitening, natural product, phenolics, flavonoids

## Abstract

**Context:**

Bambara groundnut (BG), originally from Africa, is widely distributed in Asian countries, especially in southern Thailand, and is used for food and functional foods. There is no report on the use of BG for ethnomedicine or cosmetics.

**Objective:**

To investigate collagen biosynthesis stimulation and anti-melanogenesis of the BG extracts.

**Materials and methods:**

The hulls (H) and seeds (S) of BG were collected from Trang province, Thailand and extracted by Soxhlet (S) and maceration (M) using ethanol, and boiled with distilled-water (B). Total phenolic (TPC) and total flavonoid (TFC) contents were quantified. The three antioxidant and tyrosinase inhibition activities were determined by DPPH, FIC and FTC; and the modified dopachrome methods, respectively. The collagen biosynthesis and the anti-melanogenesis activities were investigated by Sirius-Red and the melanin content assay.

**Results:**

The yields of BG extracts ranged from 1.72% to 9.06%. The BG-SS extract gave the highest TPC and TFC. The BG-HM extract showed the highest antioxidant activities (SC_50_ of 0.87 ± 0.02 mg/mL, MC_50_ of 1.83 ± 0.09 mg/mL and LC_50_ of 0.70 ± 0.06 mg/mL), tyrosinase inhibition activity (IC_50_ of 0.45 ± 0.23 mg/mL), and anti-melanogenesis activities (72.9 ± 0.08%), whereas the BG-SB extract exhibited the highest stimulation of collagen biosynthesis (18.04 ± 0.03%). All BG extracts at 0.1 mg/mL showed no cytotoxicity on human dermal fibroblasts.

**Discussion:**

The biological activities of BG extracts might be from their phytochemicals, especially phenolic and flavonoid contents.

**Conclusion:**

The BG-HB and BG-HM extracts might be promising novel active sources for anti-aging and whitening cosmeceuticals.

## Introduction

Bambara groundnut (*Vigna subterranea* (L.) Verde. [Fabaceae]) (BG) is originally from Africa, but it is widely distributed in Asian countries including Thailand, Indonesia and Malaysia. Its ability to withstand high temperatures and drought makes it a valuable crop in areas of low rainfall. BG has been widely used as a functional ingredient in the food industry, such as in the production of puddings (Ndidi et al. 2014), milk (Murevanhema and Jideani 2015) and snacks (Oyeyinka et al. 2018) due to its balance of carbohydrates, protein and fat, as well as having high amounts of particular amino acids, such as methionine and fatty acids (Bamshaiye et al. 2011; Arise et al. 2015). Besides being a food crop, other reports have shown that protein hydrolysate from Bambara groundnut exhibits antioxidant properties (Arise et al. 2016). To further increase the utilization of Bambara groundnut, it is important to explore new uses of this plant that go beyond its traditional usage, especially for cosmeceuticals.

Currently, naturally occurring constituents from plants have received much attention as an alternative source of raw materials for cosmetic products. Phytochemical screening of some of these plants shows the presence of several bioactive compounds including flavonoids, phenols, phenolic glycosides, saponins, tannins, xanthones, alkaloids and terpenoids saponins, justifying their potential use for antioxidants, anti-aging and anti-melanogenesis (Arung et al. 2011; Bourgeois et al. 2016; Kolakul and Sripanidkulchai 2017). Radical oxygen species (ROS) are chemically reactive molecule containing oxygen such as hydroxyl radical, peroxide and superoxide radicals. These molecules can cause severe oxidative damage to dermal collagen and elastic fibres, leading to skin aging. The use of antioxidants can counter those free radicals and retard the skin aging process (Li et al. 2018). In melanogenesis, tyrosinase is a key regulating enzyme that catalyses melanin synthesis within melanocytes (Chang 2012). Thus, the down-regulation of tyrosinase becomes the most prominent approach for anti-melanogenesis. Collagen Type I and Type III play a role in maintaining skin structure. During aging, dermal fibroblasts cannot produce collagen properly, and also can be destroyed by ROS, leading to a decrease in the collagen level; the collagen fibres begin to cross-link, resulting in loss of skin elasticity (Shoulders and Raines 2009). Thus, synthesis of collagen fibres from fibroblasts is essential for healthy and firm skin.

Several studies have demonstrated the effect of many natural extracts on collagen biosynthesis and anti-melanogenesis, such as Boonpisuttinant et al. (2014) who reported that the leave extract from Star grass showed the highest anti-melanogenesis on murine malanoma (B_16_F_10_) cells and stimulation of collagen biosynthesis on human dermal fibroblasts. Nagai et al. (2010) showed that isosaponarin from wasabi leaf stimulated the collagen Type I synthesis by up-regulated TGF-β type II receptor (TβR-II) and prolyl 4-hydroxylase (P_4_H) production on human skin fibroblasts.

This study investigates phytochemical constituents and the potential efficacy of Bambara groundnut (*V. subterranea*) in protecting against free radicals, metal ion, lipid peroxidation, enzyme tyrosinase, as well as anti-melanogenesis and collagen biosynthetic activity along with its cytotoxicity in order to evaluate its potential uses in cosmetic and cosmeceutical applications.

## Materials and methods

### Chemicals

Ascorbic acid (vitamin C), α-tocopherol (vitamin E), ammonium thiocyanate (NH_4_SCN), 2,2-diphenyl-1-picrylhydrazyl (DPPH), tyrosinase from mushroom, tyrosine linoleic acid, thiazolyl blue tetrazolium bromide (MTT), Folin-Ciocalteu reagent, gallic acid, quercetin and direct red 80 (Sirius red) were purchased from Sigma Aldrich (St. Louis, Missouri, USA). Dulbecco’s modified eagle medium (DMEM), foetal bovine serum (FBS) and penicillin/streptomycin were purchased from Gibco-Invitrogen (Waltham, Massachusetts, USA). Kojic acid, picric acid and dimethyl sulfoxide (DMSO) were obtained from Sisco Research Laboratories (SRL) (Maharashtra, India). Ferrous chloride (FeCl_2_), ferric chloride (FeCl_3_), aluminium chloride (AlCl_3_), sodium hydroxide (NaOH) were all acquired from VWR Chemicals BDH, Radnor (Pennsylvania, USA). Ethylenediaminetetraacetic acid (EDTA) was from Global Chemie (Mumbai, Maharashtra, India). Ferrozine was from TCI, Chuo-ku, Tokyo, Japan. All solvents were analytical grades.

### Extractions of bambara groundnut

The hulls (H) and seeds (S) of Bambara groundnut (BG) were obtained from Trang province, Thailand during September 2017. They were washed with tap water, dried at 55 °C for 48 h, and then ground into powder before the extraction. The different extraction processes as temperature and solvents may exhibit their different biological activities. The extracts were therefore made in three different methods as follows: (1) Soxhlet extraction (S), the powders of BG seeds or hulls were added into a cellulose extraction thimble which was placed inside the Soxhlet extractor. The solvent vessel was filled with 95% (v/v) ethanol at the ratio of 1:10 at 60 ± 5 °C for 24 h. The maceration extraction (M) (2), the powders were soaked with 95% (v/v) ethanol (1:10) at 37 °C for 48 h in the shaker, and (3) boiling (B), the powders were boiled at 90 ± 5 °C with distilled water (1:10) for 2 h. The extracts were filtered through filter paper no. 1. After solvent evaporation, the dried extracts were kept in an amber bottle at 4 °C. The percentage of yield was calculated as:

(1) $$\%\mathrm{Yield}of\mathrm{the}\mathrm{extract}=\frac{\mathrm{Weight}ofBG\mathrm{extract}(g)}{\mathrm{Weight}ofBG\mathrm{powder}\mathrm{before}\mathrm{extraction}(g)}\times 100$$

### Total phenolic content

The total phenolic content (TPC) was determined by Folin-Ciocalteu assay as previously described by Boonpisuttinant et al. (2014). Briefly, the plant extracts or gallic acid (50 µL), 0.5 mg/mL of Folin-Ciocalteu reagent in ethanol (75 µL) and 7.5% of Na_2_CO_3_ (75 µL) were added in a 96-well microplate and incubated at RT for 90 min. The absorbances were measured by a microplate reader 725 nm. The distilled water was used as a blank control. The amounts of phenolic contents in the extracts were calculated from the standard curve of gallic acid at various concentrations. The results were expressed as mg gallic acid equivalents (GAE)/g extract.

### Total flavonoids content

The total flavonoid content (TFC) was measured by the modified aluminium chloride colorimetric assay as previously described by Chandra et al. (2014). The BG extracts or quercetin (50 µL), 5% of NaNO_2_ (100 µL) and 10% of AlCl_3_ (25 µL) were added into 96-well microplate. After 5 min, 25 µL of 1 M NaOH was added and incubated for 10 min. Absorbances were measured by a microplate reader 450 nm. The distilled water was used as blank control. The amounts of phenolic contents in the extracts were calculated from the standard curve of quercetin at various concentrations. The results were expressed as mg quercetin equivalents (QE)/g extract.

### Antioxidant activities

#### Free radical scavenging activity

The free-radical scavenging activity of the BG extracts was measured using the DPPH assay as previously described by Boonpisuttinant et al. (2016). The BG extracts were prepared at various concentrations. The plant extracts (100 µL) and their control were added to 100 µL of 0.1 mg/mL of ethanol DPPH solution in 96 well microplates. After incubation at room temperature in the dark for 30 min, the absorbance was measured using a spectrophotometer at 515 nm. Ascorbic acid was used as a positive control. The percentage of DPPH free-radical scavenging activity of the extracts was calculated according to the following formula:

(2) $$\%\;\text{Radical}\;\text{scavenging}\;\text{activity}=\left[A_{0}-A_{1}/A_{0}\right]\times 100$$

where A_0_ is the absorbance of the control and A_1_ is the absorbance of the treated sample. The concentrations providing 50% scavenging (SC_50_) were calculated from the graph plotted between the % free radical scavenging and the sample concentrations.

#### Metal chelating activity

The metal chelating activity of the BG extracts was measured as previously described by Boonpisuttinant et al. (2016). The extracts at various concentrations were added to the solution of 1 mg/mL of FeCl_2_ (50 µL) in 1% HCl. The reaction was initiated by the addition of 50 µL of 0.1 mg/mL of ferrozine in 1% HCl. The mixture was shaken vigorously and left at room temperature, in the dark for 60 min. Absorbance of the mixture was measured by a spectrophotometer at 570 nm. EDTA was used as a positive control. All determinations were performed in triplicate (n = 3). The percentage of metal chelation activity was calculated using the following formula:

(3) $$\%\;\text{Metal}\;\text{chelating}\;\text{activity}=\left[A_{0}-A_{1}/A_{0}\right]\times 100$$

where A_0_ is the absorbance of the control and A_1_ is the absorbance of the treated sample. The concentrations providing 50% chelation (MC_50_) were calculated from the graph plotted between the % metal chelation activity and the sample concentrations.

#### Lipid peroxidation inhibition

The lipid peroxidation activity of the BG extracts was assayed by the modified Ferric-thiocyanate method as previously described by Boonpisuttinant et al. (2016). An amount of 50 µL of the samples was added to 50 µL of linoleic acid in 50% DMSO. The reaction was initiated by the addition of 50 µL of NH_4_SCN (5 mM) and 50 µL of FeCl_2_ (2 mM). The absorbance was measured at 490 nm by a microplate reader after 60 min of the reaction at 37 °C. Vitamin E was used as a standard. The percentages of lipid peroxidation inhibition of linoleic acid were calculated by the following equation:

(4) $$\%\;\text{Lipid}\;\text{peroxidation}\;\text{inhibition}=\left[\left(A_{0}-A_{1}\right)/A_{0}\right]\times 100$$

where A_0_ is the absorbance of the control and A_1_ is the absorbance of the samples. The concentrations providing 50% inhibition of lipid peroxidation (LC_50_) were calculated from the graph plotted between the % inhibition of lipid peroxidation and the sample concentrations.

### Tyrosinase inhibition activity

The tyrosinase inhibition activity of the BG extracts was performed by the modified dopachrome method using tyrosine as a substrate as previously described (Boonpisuttinant et al. 2014). Briefly, 50 µL of the samples at various concentrations, 50 µL of 0.1 mg/mL L-tyrosine, 50 µL of 0.1 mg/mL mushroom tyrosinase and 50 µL of 0.1 mM phosphate buffer were added in 96-well microplates. Kojic acid was used as a standard. The mixture was incubated at 37 °C for 60 min. Before and after incubations, the absorbances were measured at 450 nm by a microplate reader. The percentages of tyrosinase inhibition were calculated according to the following equation:

(5) % Tyrosinase inhibition activity=[(A−B)−(C−D)]/(A−B)×100

where A is the absorbance of the blank after incubation, B is the absorbance of the blank before incubation, C is the absorbance of the samples after incubation and D is the absorbance of the samples before incubation. The concentrations providing 50% inhibition (IC_50_) were calculated from the graph plotted between % inhibition activity and the concentrations.

### Cytotoxicity test

The BG extracts at various concentrations were tested for cytotoxicity on human skin fibroblasts and murine melanomas by the MTT assay as previously described by Boonpisuttinant et al. (2016). The objective of this study was to evaluate the appropriate concentration of the extracts that gave more than 80% cell viability in order to use them for collagen biosynthesis and anti-melanogenesis assays.

The human dermal fibroblasts cells and the murine melanoma (B_16_F_10_) cells were obtained from American Type Culture Collection (ATCC) (Virginia, USA). The cells were cultured under the standard conditions in the Dulbecco’s Modified Eagle Medium (DMEM) and supplemented with 10% foetal bovine serum (FBS), 100 IU/mL of penicillin and 100 µg/mL of streptomycin. Briefly, 1 × 10^4^ cells of cells were seeded into each well of 96-well plates, adjusted to 180 µL with DMEM, and incubated at 37 °C under 5% CO_2_ atmosphere for 24 h. Then, the cells were treated with 20 µL of the extracts and incubated at 37 °C under 5% CO_2_ atmosphere for 24 h. After incubation, the medium was removed, and the cells were washed with phosphate buffer saline for once. Then, 100 µL of 0.5 mg/mL MTT solution was added into each well and further incubated for 4 h. After incubation, the MTT solution was removed and 100 µL of DMSO was added to dissolve the blue-violet crystals. The plates were shaken at 200 rpm for 15 min and the absorbance at 560 nm was measured by a microplate reader. The percentage of cell viability was calculated by comparison to 100% viability of untreated cells (control) as the following:

(6) $$\%\;\text{Cell}\;\text{viability}=\left[A560_{\text{Sample}}/A560_{\text{Control}}\right]\times 100$$

where A560_Sample_ is absorbance at 560 nm of treated cells and A560_Control_ is absorbance at 560 nm of untreated cells.

### Collagen biosynthesis stimulation on human skin fibroblasts

The collagen biosynthesis was performed according to the previously described method by Boonpisuttinant et al. (2014). Briefly, 5 × 10^5^ of human skin fibroblasts were seeded into 6-well plates with the final volume of 1.8 mL, and incubated in CO_2_ incubator at 37 °C for 24 h. Then, 200 µL of the extracts at the proper concentration was added and incubated for 24 h. Vitamin C was used as a positive control. Then, 1 mL of 0.1% (w/v) Sirius red solution in saturated picric acid was added in the plate, and incubated at room temperature for 1 h. The dye was removed, and the plate was washed with 1 mL of 10 mM HCl 5 times. Then, 1 mL of 0.1 M NaOH was added to dissolve the dye. The solution was read the absorbance at 540 nm by a microplate reader. The collagen amount was determined in comparison to the control. The percentage of the collagen content was calculated as the following:

(8) % Collagen biosynthesis=(Ct/Cc)×100

where Ct was the collagen content of the treated samples and Cc was the collagen content of the control.

### Anti-melanogenesis activity on B_16_F_10_ murine melanomas

The melanin content was measured according to the previously described method by Boonpisuttinant et al. (2014). Briefly, the cells at the density of 5 × 10^5^ cells/well were plated in 6-well plates with the final volume of 1.8 mL, and incubated in CO_2_ incubator at 37 °C for 24 h. Then, 200 µL of the extract at the proper concentration was added and incubated for 72 h. Kojic acid was used as a positive control. The cells were then washed with 1X PBS, dissolved in 500 µL of 10% NaOH, and incubated at 60 °C for 1 h. The absorbance was measured at 450 nm using a microplate reader, and the melanin amount was determined in comparison with the control. The percentages of the anti-melanogenesis were calculated as the following:

(8) % Anti−melanogenesis=100−[(Mt/Mc)×100]

where Mt was the melanin content of the treated samples and Mc was the melanin content of the control.

### Statistical analysis

All determinations were performed in triplicate (n = 3). Data were presented as mean ± S.D. Statistical differences between the control and treated groups were tested by ANOVA with Tukey test. The differences were considered significant at *p* < 0.05. The relationship of the bioactivities was analyzed by Pearson’s correlation coefficient (two-tailed significance test).

## Results and discussion

### Extraction yields, phytochemical constituents, and total phenolic and flavonoid content of the BG extracts

The extraction yields of BG extracts were shown in Table 1. The BG-SS extract showed the highest extract yield of 9.09 ± 0.15%. The difference of extraction yield depends upon the different extraction techniques (Kumari et al. 2011; Murugan and Parimelazhagan 2014). The Soxhlet method found to have higher extraction yield compared to the maceration and boiling method, this may be due to its high operational temperature, solvent recycle and solvent/solute interactions (Abdolshahi et al. 2015).

**Table 1.** Extraction yields of BG extracts.

Extracts	Part used	Extraction methods	% Yields
BG-HS	Hull	Soxhlet	8.45 ± 0.41
BG-HM	Hull	Maceration	1.51 ± 0.24
BG-HB	Hull	Boiling	5.05 ± 0.24
BG-SS	Seed	Soxhlet	9.09 ± 0.15
BG-SM	Seed	Maceration	4.72 ± 0.20
BG-SB	Seed	Boiling	2.76 ± 0.23

The data are expressed as mean ± SD. BG is the Bambara groundnut; HS is the hull extracts prepared by Soxhlet with EtOH; SS is the seed extracts prepared by Soxhlet with EtOH; HM is the hull extracts prepared by maceration with EtOH; SM is the seed extracts prepared by maceration with EtOH, HB is the hull extracts prepared by boiling with distilled water and SB is the seed extracts prepared by boiling with distilled water.

**Figure 1.**
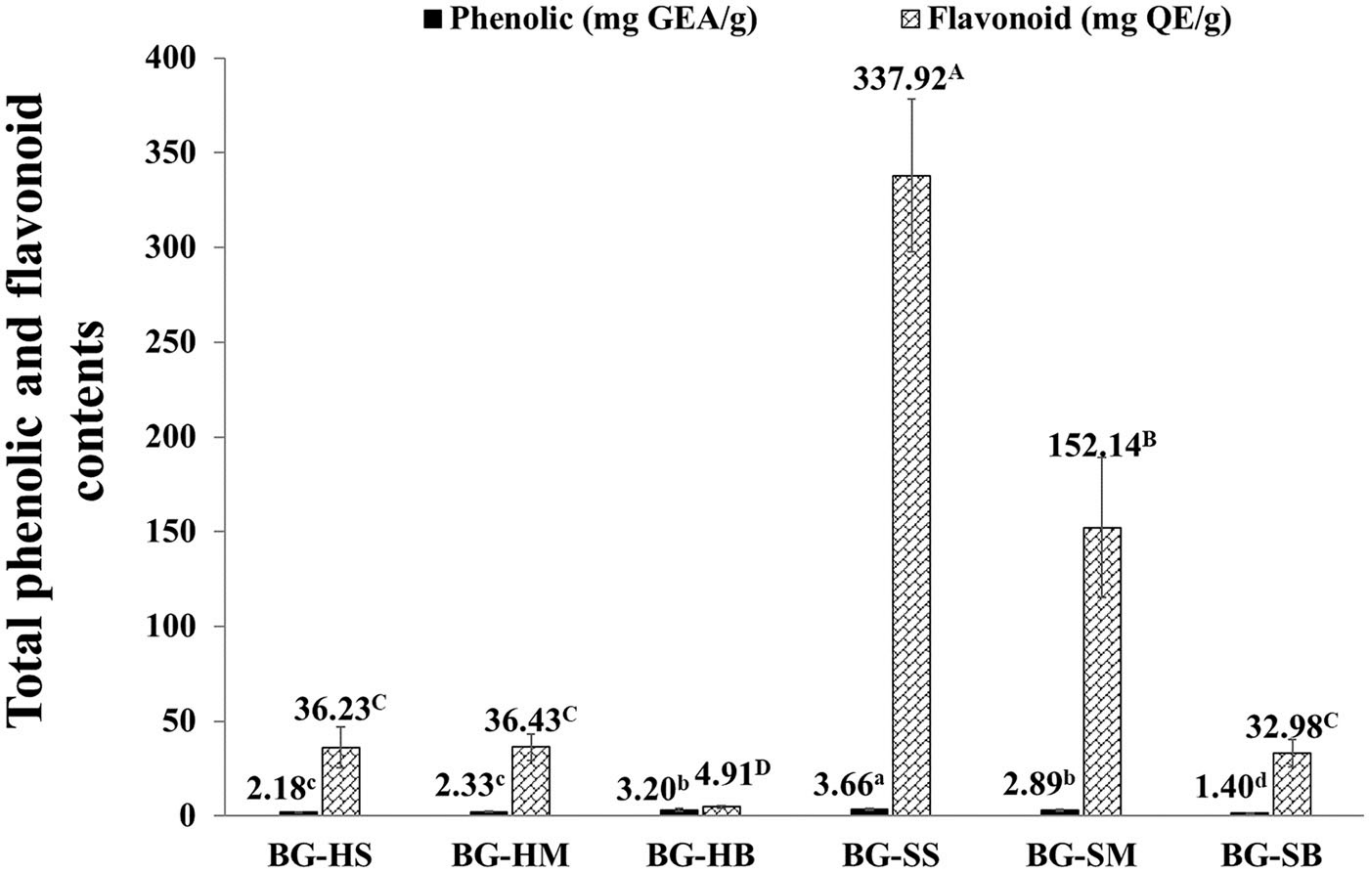
Total phenolic content (TPC) (mg GAE/g) and total flavonoid content (TFC) (mg QE/g) of BG extracts. The data are expressed as mean ± SD and different superscript letters (^a–d^ for TPC and ^A–C^ for TFC) in the column indicate denote significant differences at *p* < 0.05 – Tukey test. BG is the Bambara groundnut; HS is the hull extracts prepared by Soxhlet with EtOH; SS is the seed extracts prepared by Soxhlet with EtOH; HM is the hull extracts prepared by maceration with EtOH; SM is the seed extracts prepared by maceration with EtOH, HB is the hull extracts prepared by boiling with distilled water and SB is the seed extracts prepared by boiling with distilled water.

The total phenolic content (TPC) and total flavonoid content (TFC) were measured by Folin-Ciocalteu assay and modified aluminium chloride colorimetric assay, respectively (Figure 1). The BG-SS extract was exhibited in both the highest total phenolic compounds (3.66 ± 0.40 mg GAE/g extract) and the flavonoid contents (337.92 ± 40.50 mg QE/g extract**)**. In this study, the Soxhlet extraction was also found that the extraction yield and flavonoid content gave more than other methods. However, the phenolic and flavonoid compounds in BG extracts did not significantly correlate to their extraction yields. Phenolic and flavonoid compounds are plant secondary metabolites, and the largest category of phytochemicals constituents of plant extracts which exhibit interesting bioactivities, such as antioxidant, anticancer, anti-inflammatory, anti-diabetes and antimicrobial activities (Panche et al. 2016; Shahidi and Yeo 2018). Thus, the presence of total phenolic compounds and total flavonoids content in BG extracts might be responsible for many bioactivities. The reports from Olaleye et al. (2013) and Harris (2019) showed the seed extracts from BG have been reported high content of flavonoids and tannins, especially Testa part (0.79 mg/100 g and 0.84 mg/100 g, respectively), and show potential antioxidant and antimicrobial activities. As previously reported, the ethanol extract from *Croton roxburghii* (Euphorbiaceae) had a phenolic content and flavonoid content of about 19.41 mg GAE/g and 7.54 mg QE/g, respectively, which was also inhibited cellular melanin content and tyrosinase activity in α-MSH-stimulated B_16_F_10_ cells (Chatatikun and Chiabchalard 2017; Chatatikun et al. 2019).

### Antioxidant and tyrosinase inhibition activities of the BG extracts

This present study revealed that all BG extracts demonstrated antioxidant activities including free radical scavenging activity, metal chelating and inhibition of lipid peroxidation activities, and also tyrosinase inhibition activity (Table 2). The BG-HM and BG-SB extracts gave the highest free radical scavenging activity with the SC_50_ value of 0.87 ± 0.02 and 0.92 ± 0.15 mg/mL respectively, but lower than that of ascorbic acid, a strong radical scavenger (SC_50_ value of 0.04 ± 0.00 mg/mL) of about 22-fold. The BG-HB and BG-SB extracts gave the highest metal chelating activity with the MC_50_ values of 0.88 ± 0.16 and 0.63 ± 0.04 mg/mL respectively, which were comparable to EDTA, a metal chelator (MC_50_ value of 0.73 ± 0.08 mg/mL). For the lipid peroxidation inhibition activity, the BG-HM and BG-HB extracts gave the highest activity with the LC_50_ value of 0.70 ± 0.06 and 0.83 ± 0.20 mg/mL, which was comparable to that of α-tocopherol, a standard antioxidant, (LC_50_ value of 0.61 ± 0.06 mg/mL). Previously, Nyau et al. (2015) showed that brown and red Bambara groundnut exhibited the highest DPPH free radical scavenging activity EC_50_ value of 0.347 ± 0.042 mg/mL and 0.495 ± 0.012 mg/mL, respectively. Further, *Cassia tora* (Leguminosae) methanol extract had the highest phenolic contents (287.73 ± 0.16 mg GAE/g), flavonoid content (37.86 ± 0.53 mg QE/g), and DPPH radical scavenging (IC_50_ value of 9.898 µg/mL) (Aryal et al. 2019). Moreover, Huyut et al. (2017) reported the flavonoids and phenolic compound such as oenin, malvin, arachidonoyl dopamine, callistephin, silychristin and 3,4-dihydroxy-5-methoxybenzoic acid exhibited more effective antioxidant activity (Fe^2+^ metal chelating, Fe^3+^ and Cu^2+^ reduction and free radical scavenging) than that observed for the positive control.

**Table 2.** Antioxidant and tyrosinase inhibition activities of BG extracts.

Extracts	Antioxidant activities			Tyrosinase inhibition activity (IC_50_ mg/mL)
	Free radical scavenging activity (SC_50_ mg/mL)	Metal chelating activity (MC_50_ mg/mL)	Lipid peroxidation inhibition (LC_50_ mg/mL)	
BG-HS	1.83 ± 0.16^e^	7.21 ± 0.76^D^	3.79 ± 0.38^ii^	0.07 ± 0.07^I^
BG-HM	0.87 ± 0.02^b^	1.83 ± 0.09^B^	0.70 ± 0.06^i^	0.45 ± 0.23^II^
BG-HB	1.18 ± 0.08^c^	0.88 ± 0.16^A^	0.83 ± 0.20^i^	1.46 ± 0.30^II,III^
BG-SS	1.41 ± 0.09^d^	3.39 ± 0.53^C^	4.68 ± 0.36^ii^	0.84 ± 0.27^II^
BG-SM	2.09 ± 0.06^e^	7.23 ± 0.32^D^	3.56 ± 0.94^ii^	0.64 ± 0.27^II^
BG-SB	0.92 ± 0.15^b,c^	0.63 ± 0.04^A^	6.93 ± 1.05^iii^	2.86 ± 0.69^III^
Ascorbic acid	0.04 ± 0.00^a^	N.D.	N.D.	–
α-Tocopherol	–	N.D.	0.61 ± 0.06^i^	–
EDTA	–	0.73 ± 0.08^A^	–	–
Kojic acid	–	–	–	0.05 ± 0.03^I^

The data are expressed as mean ± SD and different superscript letters (^a–e^ for SC50, ^A–C^ for MC50, ^i–iii^ for LC and ^I–III^ for IC) in the column indicate denote significant differences at *p* < 0.05 – Tukey test. BG is the Bambara groundnut; HS is the hull extracts prepared by Soxhlet with EtOH; SS is the seed extracts prepared by Soxhlet with EtOH; HM is the hull extracts prepared by maceration with EtOH; SM is the seed extracts prepared by maceration with EtOH, HB is the hull extracts prepared by boiling with distilled water and SB is the seed extracts prepared by boiling with distilled water.

The tyrosinase inhibition activities of the BG extracts are also shown in Table 2. The highest tyrosinase inhibition activity was found in the BG-HS extract with the IC_50_ value of 0.07 ± 0.07 mg/mL, which was comparable to kojic acid, a strong tyrosinase inhibitor (IC_50_ value of 0.05 ± 0.03 mg/mL). Previously studied, the structure of tannins was reported to contain a benzene ring and many hydroxyl groups, which is very similar to the construction of tyrosinase substrate, and might inhibit the oxidation process of tyrosinase-catalyzed and showed greater inhibition on the tyrosinase activity than arbutin (Chai et al. 2015, 2018; Deng et al. 2016). Xanthones are a class of polyphenolic compounds comprising of tricyclic aromatic ring and several different substituents such as isoprene, methoxy and phenyl groups, aromatic protons, phenolic hydroxyl groups, hydroxyl protons and dihydrofuran rings (Shan et al. 2011). In addition, it has been demonstrated that polyphenol can form stable metal complexes through their hydroxyl groups (Psotová et al. 2003) which may relate to a decrease in activity of copper containing enzyme including tyrosinase. Şöhretoğlu et al. (2018) reported the flavonoids, especially those containing 3′,4′-dihydroxyl substitution on ring B significantly increase the tyrosinase inhibitory effect, were more potent than kojic acid. Moreover, Zolghadri et al. (2019) explains that the C-6 and C-7 hydroxyl groups of isoflavone might play a role in tyrosinase inhibitory activity. Therefore, the antioxidant and tyrosinase inhibition activities of BG extracts might be due to the synergistic effect of polyphenol contents including flavonoids and phenolic compounds.

### Cytotoxicity of the BG extracts

**Figure 2.**
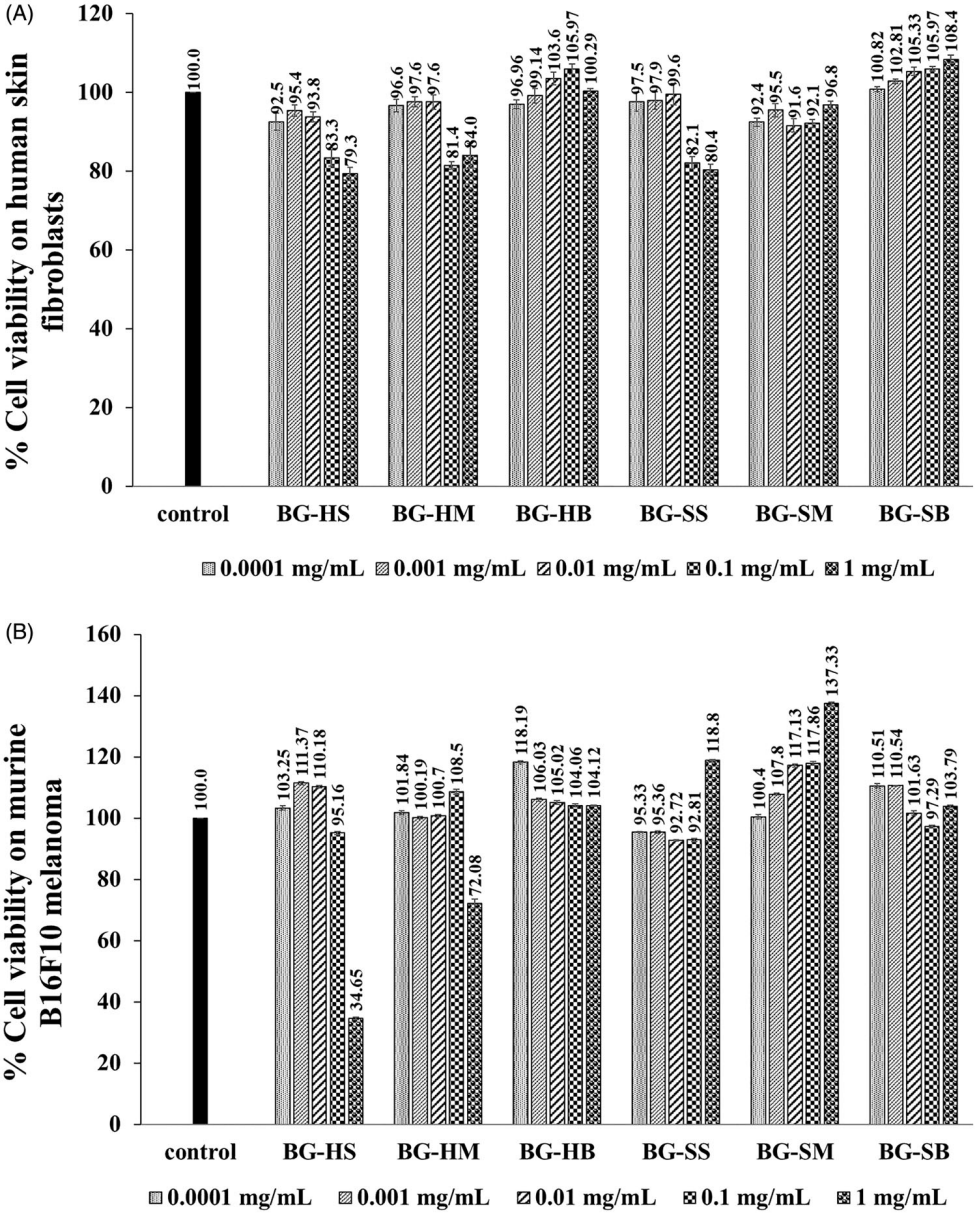
Cytotoxicity of BG extracts at various concentrations on human skin fibroblasts (A) and murine melanoma (B_16_F_10_) cells (B). BG is the Bambara groundnut; HS is the hull extracts prepared by Soxhlet with EtOH; SS is the seed extracts prepared by Soxhlet with EtOH; HM is the hull extracts prepared by maceration with EtOH; SM is the seed extracts prepared by maceration with EtOH, HB is the hull extracts prepared by boiling with distilled water and SB is the seed extracts prepared by boiling with distilled water.

The cytotoxicity of the hull and seed extracts of BG at 0.1 mg/mL concentrations on human dermal fibroblasts (Figure 2(A)) and murine melanoma (B_16_F_10_) cell line (Figure 2(B)) exhibited no cytotoxicity, since they gave more than 80% cell viability when compared with the control (Buapool et al. 2013). However, the higher concentration (1 mg/mL) of almost BG extracts is considered as slightly cytotoxic except the BG-HS extract which was dramatically cytotoxic to B_16_F_10_ cells. Therefore, the concentration of 0.1 mg/mL of all BG extracts has been considered for investigation of the collagen biosynthesis on human dermal fibroblasts and anti-melanogenesis on B_16_F_10_ cells. The different cytotoxic effect of the extracts might depend on the cell types which exhibit difference responses towards a specific plant extract (Rezk et al. 2015). Thus, the results have been ensured that the reduction of the melanin content and stimulation of collagen will not be due to cell death. On the other hand, the result of cytotoxicity on human dermal fibroblasts indicated no toxicity for skin application at the BG concentration of 0.1 mg/mL.

### Collagen biosynthesis stimulation of the BG extracts

**Figure 3.**
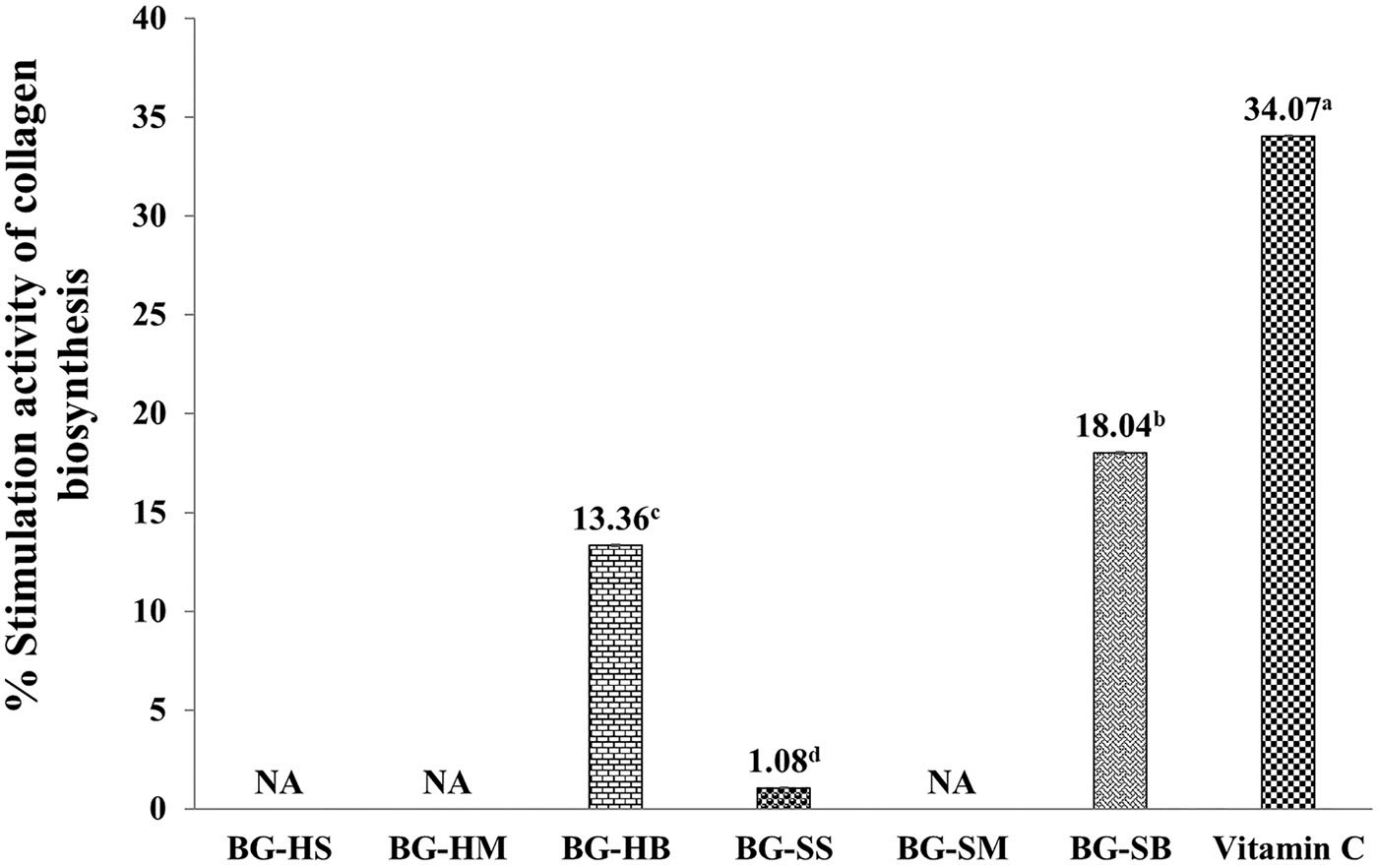
Collagen biosynthesis stimulation on human skin fibroblasts of BG extracts at concentration of 0.1 mg/mL. The data are expressed as mean ± SD and different superscript letters (^a–d^) in the column indicate denote significant differences at *p* < 0.05 – Tukey test. BG is the Bambara groundnut; HS is the hull extracts prepared by Soxhlet with EtOH; SS is the seed extracts prepared by Soxhlet with EtOH; HM is the hull extracts prepared by maceration with EtOH; SM is the seed extracts prepared by maceration with EtOH, HB is the hull extracts prepared by boiling with distilled water and SB is the seed extracts prepared by boiling with distilled water.

Environmental factors, nutrition, alcohol abuse and smoking are risk factors to stimulate the loss of skin elasticity, wrinkled and flaccid skin. Likewise, plant phenolic compounds are able to maintain skin homogeneity, stimulation of skin cell renewal, elastin and collagen (Dzialo et al. 2016). Normally, collagen is digested with metalloproteinases (MMPs), often called collagenases. Likewise, the calcium ions (Ca^2+^) and Zinc ions (Zn^2+^) are required metal cofactors for stability and activity of collagenase (Eckhard et al. 2013). Following the cytotoxicity result above, the proper concentration for investigation of collagen biosynthesis stimulation was 0.1 mg/mL since it showed no cytotoxicity on human dermal fibroblasts of BG extracts (Figure 2(A)). The percentages of collagen biosynthesis stimulation of Bambara groundnut extracts were shown in Figure 3.

The BG-SB extract exhibited the highest stimulation of collagen biosynthesis on human dermal fibroblasts (18.04 ± 0.03%) when compared to the control (no treatment) (*p < 0.05*), whereas ascorbic acid, a collagen stimulator, gave 34.07 ± 0.03%. The collagen stimulation of the BG-SB extract was significantly lower than that of ascorbic acid by about twofold, whereas the BG-HB and BG-SS extracts showed lower stimulation activity of 13.36 ± 0.03%, and 1.08 ± 0.03%, respectively. Some phenolic and flavonoid compounds may be responsible for the highest activity on BG-SB extract. The formula herbal extract (PH) mixed with *Equisetum arvense* (Equisetaceae), *Achillea millefolium* (Asteraceae), *Echinacea purpurea* (Asteraceae) and *Hyssopus officinalis* (Lamiaceae) was found to have the highest phenolic compound (chlorogenic acid, caffeic acid, luteolin and apigenin) and exhibited stimulation of collagen synthesis on L929 Mouse fibroblasts cell line (Alexandru et al. 2015). Recently, polyphenols present in plants such as gallic acid, elaeocarpusin, pedunculagin and ellagitannin enhanced proliferation, collagen production and inhibition of collagenase and elastase activity in the fibroblasts (Dzialo et al. 2016). Therefore, the effect of collagen stimulation of BG-SB might be caused by decreasing activity of collagenase of a metalloproteinase enzyme.

### Anti-melanogenesis activity of the BG extracts

**Figure 4.**
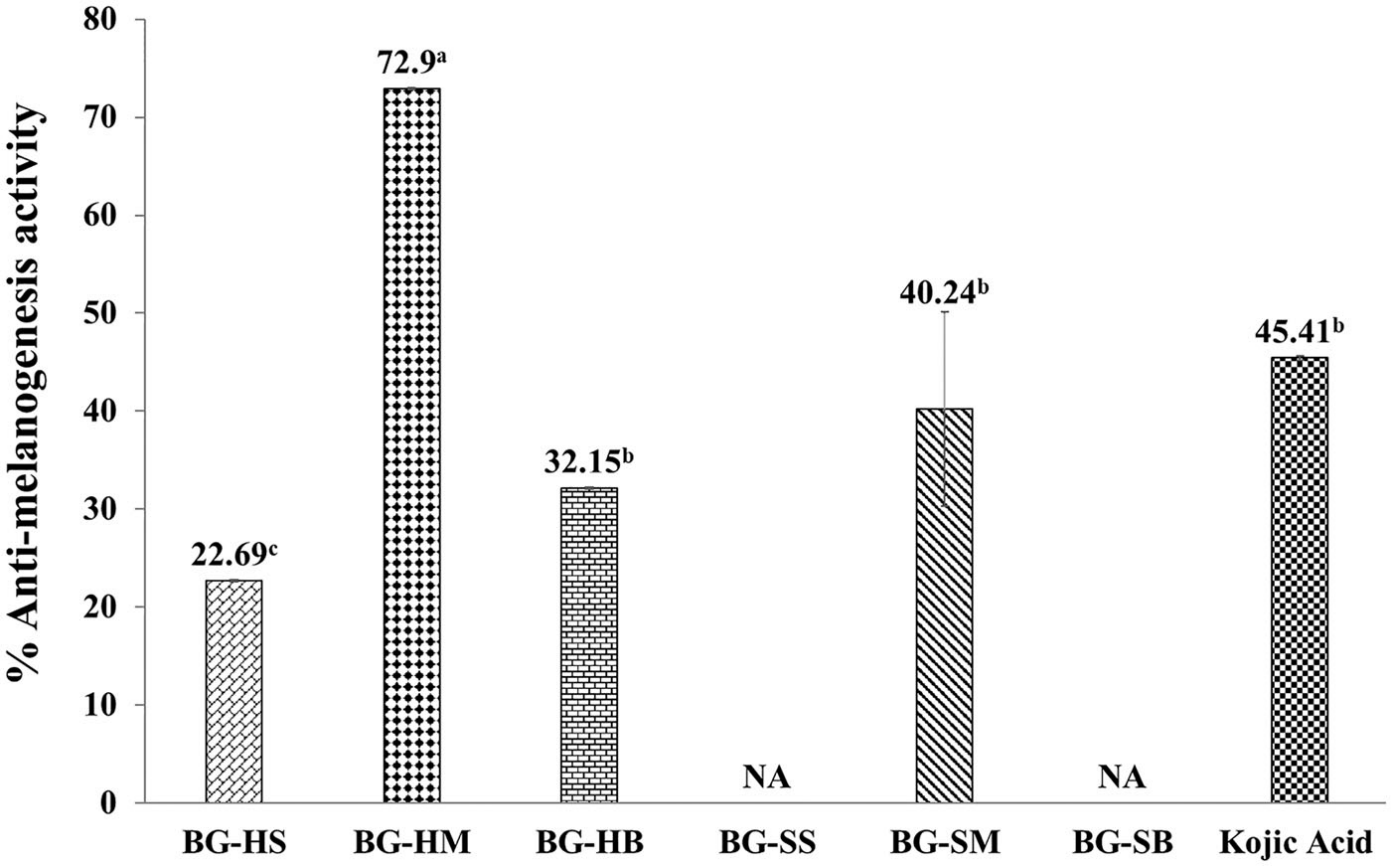
Anti-melanogenesis on murine melanomas (B_16_F_10_) cell line of BG extracts at concentration of 0.1 mg/mL. The data are expressed as mean ± SD and different superscript letters (^a–c^) in the column indicate denote significant differences at *p* < 0.05 – Tukey test. BG is the Bambara groundnut; HS is the hull extracts prepared by Soxhlet with EtOH; SS is the seed extracts prepared by Soxhlet with EtOH; HM is the hull extracts prepared by maceration with EtOH; SM is the seed extracts prepared by maceration with EtOH, HB is the hull extracts prepared by boiling with distilled water and SB is the seed extracts prepared by boiling with distilled water.

Melanogenesis is a complex process for melanin pigment production in malanosomes of melanocytes, which respond to ultraviolet B (UVB)-irradiation. Also, it plays a role in protection from skin damage from ultraviolet radiation. However, overproduction of melanins can cause skin problems such as freckles, melasma and melanoma skin cancer (Pillaiyar et al. 2017). All BG extracts at the concentration of 0.1 mg/mL did not show cytotoxicity on the murine melanomas (B_16_F_10_) cell line (Figure 2(B)). Therefore, this concentration was proper to investigate anti-melanogenesis of the BG extracts. Most BG extracts at 0.1 mg/mL demonstrated the anti-melanogenesis activity on B_16_F_10_ cells, especially the BG-HM extract, which gave the highest activity with the inhibition of 72.90 ± 0.08% and was superior to that of kojic acid (45.35 ± 0.28%) by about 1.6-fold (Figure 4). The phenolic and flavonoid content in the BG-HM might be responsible for anti-melanogenesis activity, which presents their phytochemicals content in medicinal plants on B_16_F_10_ cells (Campos et al. 2013; Kim et al. 2015). Likewise, hesperidin, a flavonoid and a popular antioxidant activity, suppressed melanin synthesis by stimulated Extracellular signal-regulated kinases (Erk1/2) phosphorylation and degraded microphthalmia‑associated transcription factor (MITF) (Lee et al. 2015). Moreover, *Nymphaea nouchali* flower extract (NNFE) has compounds of seven flavonoids and two spermidine alkaloids, which significantly reduced melanogenesis and inhibited mushroom tyrosinase activity as well as reduced melanin content in vitro and in vivo by downregulating MITF expression via suppression of cAMP (Alam et al. 2018). Previously found results, the phenolic and flavonoid compound present in plants, and plant extracts inhibited cellular melanin content and tyrosinase activity in α-MSH-stimulated B_16_F_10_ cells though suppressing MITF, tyrosinase, TRP1 and TRP-2 (Zhu et al. 2016; Chatatikun et al. 2019). Therefore, the presence of radical scavenging or antioxidant activities might be related to down-regulation of melanogenesis (Huang et al. 2015).

### Correlation of the bioactivities of the BG extracts

Since there is no report about the bioactivities of BG, the results of correlation of their biological activities might define or describe their biological activities (Table 3). The correlation coefficient value is descriptors of relationship of biological activities including weak (*R* = ± 0.00 to ± 0.49), moderate (*R* = ± 0.50 to ± 0.79) or strong (*R* = ± 0.80 to ±1.00) (Schober et al. 2018). In the group of three mechanisms of antioxidation activities, which include the free radical scavenging activity (SC), the Inhibition of lipid peroxidation activity (LC) and the metal chelation activity (MC), it was demonstrated that the SC has a significant moderate correlated relationship to the MC and the LC with the Pearson correlation coefficient *(R*) of 0.707 and 0.521, respectively. Moreover, the relationship between the SC and the collagen biosynthesis activity on human dermal fibroblasts (CB) with the *R* of 0.500, and the LC and the anti-melanogenensis activity on B_16_F_10_ cells (AM) with the *R* of 0.781, were classified as moderate relationship (*R* < 0.8). Interestingly, the MC and the CB have an excellent relationship (*R* > 0.8) since they exhibited very high on the Pearson correlation coefficient (*R*) of 0.965 at a significant level (*p* value) of 0.01, which means if the metal chelation activity of the BG extracts increase, the collagen biosynthesis activity would be dramatically increased as well. It has been reported that collagenase, which is an enzyme requiring metal cofactors such as calcium ions (Ca^2+^) and Zinc ions (Zn^2+^) (Eckhard et al. 2013), could reduce its activity and stability by metal chelation. Thus, the metal chelation activity of the BG extracts might up-regulate the collagen biosynthesis on human dermal fibroblasts via inhibition of collagenase activity.

**Table 3.** Correlation of the bioactivities of the BG extracts.

Bioactivities	Correlation (R)					
	SC	MC	LC	IC	CB	AM
SC	–	0.707	0.521	−0.455	0.500	0.205
MC	0.707	–	0.149	−0.493	0.965^a^	−0.275
LC	0.521	0.149	–	−0.225	−0.009	0.781
IC	−0.455	−0.493	−0.225	–	−0.426	0.004
CB	0.500	0.965^a^	−0.009	−0.426	–	−0.430
AM	0.205	−0.275	0.781	0.004	−0.430	–

Free radical scavenging activity (SC), Metal chelating activity (MC), Lipid peroxidation inhibition (LC), Tyrosinase inhibition activity (IC) were calculated from 1/SC50 (mL/mg), 1/MC50 (mL/mg), 1/LC50 (mL/mg) and 1/IC50 (mL/mg), respectively. Collagen biosynthesis stimulation (CB) and Anti-melanogenesis activity (AM) were the % collagen biosynthesis stimulation and the % anti-melanogenesis activity.

^a^Correlation is significant at the 0.01 level (2-tailed).

However, no relationship exists between the tyrosinase inhibition activity (IC) and anti-melanogenesis activity (AM) of the BG extracts, which indicates that the suppression of melanogenesis on B_16_F_10_ cells might not be only from the inhibition of mushroom tyrosinase activity. Although, cellular tyrosinase on B_16_F_10_ cells is a key enzyme of melanogenesis by down-regulate melanogenesis including α-MSH-stimulated microphthalmia-associated transcription factor (MITF), and tyrosinase related protein-1&2 (TRP1&2), the study on mushroom tyrosinase might not relate to the *in vitro* melanogenesis study. It has been reported that *Pistacia atlantica* subsp. *mutica* extracts can significantly inhibit mushroom tyrosinase activity, but not significantly against cellular tyrosinase activity and melanin synthesis (Lee et al. 2015; Eghbali-Feriz S et al. 2018).

## Conclusions

Bambara groundnut has been used as a functional food for many years ago, since it contains a lot of nutrients such as proteins, carbohydrates and lipids. This present study demonstrated that the BG extracts gave the antioxidant activity, especially BG-HM extract, which showed the highest tyrosinase inhibition and anti-melanogenesis activity and was superior to kojic acid. Moreover, the BG-HB and BG-SB extracts exhibited the highest stimulation of collagen biosynthesis on human dermal fibroblasts. All BG extracts at 0.1 mg/mL had indicated no toxicity on skin since they showed no cytotoxicity on human dermal fibroblasts. The biological activities of the BG extracts might be from the synergistic effect of phytochemical constituents, especially phenolic and flavonoid compound. The results suggested that the BG-HB and BG-HM are a promising candidate as an anti-aging and whitening agent respectively. The other mechanisms for anti-aging and the whitening effect as well as clinical research on volunteer will be further performed for the development of cosmeceutical applications.
